# Regulation of cell proliferation and apoptosis by growth hormone during zebrafish auditory hair cell regeneration

**DOI:** 10.1186/1471-2105-13-S12-A3

**Published:** 2012-07-31

**Authors:** Gopinath Rajadinakaran, Huifang Sun, Claire Rinehart, Eric Rouchka, Michael Smith

**Affiliations:** 1 Department of Biology and Bioinformatics and Information Science Center, Western Kentucky University, Bowling Green, KY 42101, USA; 2 Department of Computer Engineering and Computer Science, University of Louisville, Louisville, KY 40292, USA

## Background

In order to develop treatments or preventive measures for auditory hair cell loss, an understanding of both the process of auditory hair cell regeneration and factors that influence this process, is needed. Our previous microarray analysis showed that growth hormone (GH) was significantly upregulated during zebrafish auditory hair cell regeneration, coupled with cell proliferation [1,2]. We further tested the effects of GH on zebrafish auditory hair cell regeneration by injecting GH after sound exposure and found that GH can efficiently promote post-trauma auditory hair cell regeneration, which may be achieved through stimulating proliferation and suppressing apoptosis [3]. In the current study, we used Next Generation Sequencing (NGS) to examine the possible GH pathways involved in zebrafish auditory hair cell regeneration.

## Materials and methods

Groups of 20 zebrafish were exposed to a 150 Hz tone at 179 dB re 1 μPa RMS for 40 h. Following acoustic exposure, the fish were injected with either phosphate buffer, GH, or a GH antagonist. In addition, one baseline group received no acoustic stimulus or injection. RNA was extracted from the inner ear tissues and cDNA was synthesized for NGS. Data was generated using the Illumina Pipeline version SCS 2.8.0 and sequence alignment was done using TopHat. The aligned reads were then annotated using Cufflink and the differential expression of transcripts was performed by Cuffdiff. Only the statistically significant reads were used in further analysis and pathways were examined using Ingenuity Pathway Analysis (IPA).

## Results and conclusions

**Figure 1:**
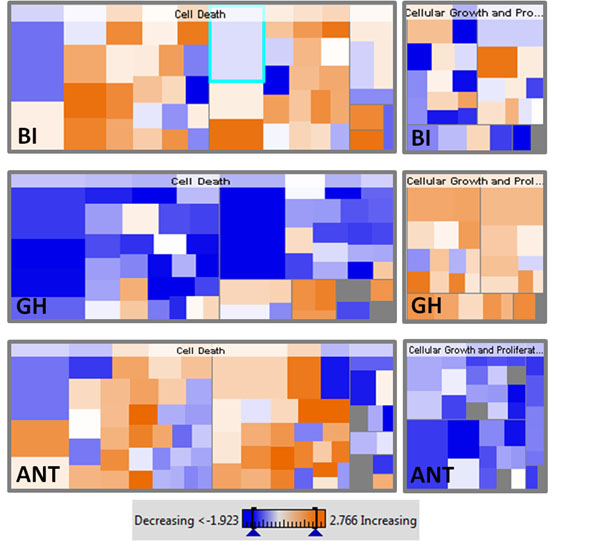
**Heat maps for cell death and cell growth/proliferation functions generated by IPA under different treatment conditions (BI-buffer injected, GH-growth hormone, ANT-antagonist) at 1 day post-sound exposure.** GH downregulates (blue) cell death pathways and upregulates (orange) cellular growth and proliferation pathways, while the opposite pattern is found for the GH antagonist.

Genes associated with cellular growth and proliferation, cell cycle, cell death, cancer, tissue development, and neurological disease were highly regulated in our data sets comparing baseline to buffer-injected fish, buffer-injected to antagonist-injected fish, and buffer-injected to GH-injected fish. Heat maps (Figure 1) and canonical signaling pathways were generated using IPA. We further examined the effect of GH by analyzing the GH receptor pathway. Interestingly, we found the JAK-STAT pathway to be highly upregulated in buffer-injected and GH-injected conditions but a strong downregulation was observed in the antagonist-injected condition. We also found the upregulation of IGF2 and downregulation of SOCS in the GH condition while the reverse was observed in the antagonist condition. These results suggest that GH influences zebrafish hair cell regeneration by promoting proliferation and suppressing apoptosis through the JAK-STAT pathway.
